# Ruan Jian Qing Mai Recipe Inhibits the Inflammatory Response in Acute Lower Limb Ischemic Mice through the JAK2/STAT3 Pathway

**DOI:** 10.1155/2022/2481022

**Published:** 2022-08-18

**Authors:** Di Zhu, Chenglin Jia, Tongkai Cai, Jiacheng Li, Xia Feng, Nan Chen, Cheng Zhao, Yuzhen Wang, Yongbing Cao, Yemin Cao

**Affiliations:** ^1^Shanghai University of Traditional Chinese Medicine, Shanghai 201203, China; ^2^Department of Vascular Diseases, Shanghai TCM-Integrated Hospital, Shanghai University of Traditional Chinese Medicine, Shanghai 200082, China; ^3^Institute of Vascular Diseases, Shanghai TCM-Integrated Hospital, Shanghai University of Traditional Chinese Medicine, Shanghai 200082, China; ^4^PLA Naval Medical University, Shanghai 200433, China

## Abstract

Ruan jian qing mai recipe (RJQM) is an empirical prescription for treating arteriosclerosis obliterans (ASO). However, the mechanism of RJQM recipe-mediated ASO attenuation has not yet been elucidated. Therefore, this study aimed to explore the mechanism by which the RJQM recipe relieves ASO in a mouse model of lower limb ischemia, which was established by ligating and breaking the femoral artery of the left lower limb. The surgical groups were divided into the ischemic group, beraprost sodium group, low-dose RJQM group, medium-dose RJQM group, and high-dose RJQM group. Normal mice were set as the control group. The blood flow of the lower limb was examined on days 7 and 14. At the end of animal procedures, blood samples were collected, and the rectus femoris of the left lower limb were harvested. Results revealed that mice in the ischemic group demonstrated low blood flow. Additionally, hematoxylin and eosin, and Masson staining results showed that inflammation of the rectus femoris was obvious in the ischemia group, and the level of fibrosis was increased. Blood flow was recovered in all treatment groups compared to the ischemic group, and the inflammatory infiltration and fibrosis of the rectus femoris were relieved after RJQM treatment. The serum levels of interleukin (IL)-17A and IL-21 were decreased, and the expression of JAK2/STAT3 proteins was inhibited in all RJQM treatment groups compared to the ischemia group. Furthermore, the improvement of IL-17A, IL-21, and rectus femoris fibrosis was more obvious with increasing treatment time. In conclusion, RJQM can effectively alleviate ASO and promote the recovery of lower limb blood flow by regulating the JAK2/STAT3 signaling pathway to reduce the inflammatory response.

## 1. Introduction

Arteriosclerosis obliterans (ASO), a manifestation of atherosclerosis in the lower limbs, mainly characterized mainly by lower extremity arterial intima thickening and lumen stenosis or occlusion, causing intermittent claudication, rest pain, ischemic ulcer, and even gangrene [1]. ASO is a common disease in the elderly, seriously affecting the patients' quality of life [2]. According to epidemiological studies [3], approximately 200 million people worldwide are currently suffering from ASO, and the prevalence significantly increases with age, affecting about 50% of the population aged ≥85 years.

Noteworthy, ASO pathogenesis is complex and still unclear. To date, studies have presented numerous theories on ASO pathogenesis, including lipid infiltration theory [4] and vascular endothelial injury theory [5]. Moreover, some studies have proposed that atherosclerosis is a chronic inflammatory disease characterized by severe immune activities, and inflammation modulates atherosclerosis occurrence and development [6, 7]. Given that inflammation is always present during the occurrence, development, and final outcome of the disease, anti-inflammatory treatment, and inhibition of inflammatory cytokines are essential [8]. Traditional Chinese medicine (TCM) has a remarkable curative effect in the treatment of inflammatory diseases. Additionally, Chinese herbal medicine has fewer side effects; thus, it can provide better choices for clinical anti-inflammatory treatment and immunotherapy of ASO.

Ruan jian qing mai recipe (RJQM), an empirical prescription for treating ASO proposed by Professor Jiuyi Xi, is composed of *Sedum sarmentosum*, *Siegesbeckia glabrescens*, Seaweeds, Oysters, etc. *Sedum sarmentosum* has been shown to eliminate vascular inflammation, regulate immunity, and induce antibacterial and anti-inflammatory effects [9]. *Siegesbeckia glabrescens* aqueous and ethanol extracts have antibacterial, anti-inflammatory, antioxidant, and immunosuppressive effects; hence, it is commonly used in clinical therapy of cardiovascular and cerebrovascular diseases [10]. Previous studies have also revealed that these Chinese anti-inflammatory medicines have good effects on atherosclerosis [11, 12], which is consistent with the theory that inflammation is closely associated with atherosclerosis.

In our previous study, we found that RJQM decoction could alleviate atherosclerotic plaque by reducing blood lipid levels of animals, accelerating lipid metabolism, and inhibiting the inflammatory response. Furthermore, it could promote the formation of collateral circulation in ischemic sites and improve blood supply [13]. However, the mechanism through which it alleviates inflammation in atherosclerosis has not yet been elucidated.

Although there is no perfect ASO animal model, the hindlimb ischemia mice model reproduces many key characteristics of ASO, such as the change of composition of the rectus femoris. Therefore, this model is widely used to explore the related potential mechanisms of ischemic limb diseases [14]. Herein, a mouse model of lower limb ischemia was established by ligating and breaking the femoral artery of the left lower limb, and then it was used to explore the anti-inflammatory effect and underlying mechanism of RJQM on ASO. The main aim of this study was to explore the anti-inflammatory effect and mechanism of RJQM on ASO, with the overarching goal of providing a theoretical foundation for clarifying the efficacy of RJQM and expanding its clinical application.

## 2. Materials and Methods

### 2.1. Experimental Animals

C57BL/6 male mice (aged five weeks; weighed 16–20 g; *n* = 120) were obtained from Shanghai Jihui Experimental Animal Feeding Co., Ltd. The animals were raised under standard conditions (constant temperature, 23–25°C; humidity, 32%–35%; and 12-h light/dark cycle), with *ad libitum* access to food and water. Notably, the experiment began one week after adaptive feeding. Mice were randomly divided into six groups: normal control group (CON group, *n* = 20); ischemia group (ISC group; *n* = 20), beraprost sodium (Beijing Taide Pharmaceutical Co., Ltd., 010022) group (BPS group; *n* = 20), RJQM (Shanghai Qingpu Liantang Pharmaceutical Co., Ltd., 20210409) low-dose group (L group; *n* = 20), RJQM medium-dose group (M group; *n* = 20), and RJQM high-dose group (H group; *n* = 20). After the establishment of the ischemia model, BPS (15.6 *μ*g/kg) and RJQM (0.462, 0.924, and 1.848 g/kg) were administered daily for 14 consecutive days. This experiment was approved by the Laboratory Animal Ethics Committee of Tongji University (TJ-HB-LAC-2021-30), and all animal experiments were performed according to the Helsinki declaration.

### 2.2. Construction of the ASO Mouse Model

One week after adaptive feeding, acute hindlimb ischemia was induced by the ligation and disconnection of the femoral artery in the left hindlimb. Briefly, mice were first anesthetized with isoflurane gas (the anesthetic concentration was 2%–3%, whereas the maintenance concentration was 1.5%–2%). Then, the limbs of the mice were fixed on the surgical platform, and the hindlimbs of the mice were depilated with a razor and hair removal ointment. After disinfection with 75% medical alcohol, the skin was longitudinally cut (a length of about 1–1.5 cm) to the subcutaneous tissue in the medial thigh of the left hindlimb of the mice. The blood vessels were then fully exposed, followed by the separation of the blood vessels and nerves and ligation and breaking of the femoral artery. Finally, bleeding was examined, and 75% alcohol was used to disinfect the wound before it was sutured. Successful modeling was confirmed by the determination of blood flow after operation: the ratio of left limb and right limb blood flow decreased to less than 30%. The mice were then put back in the cage until the stabilization of their vital signs.

### 2.3. Laser Doppler Perfusion Imaging Detection

Under the condition of continuous anesthesia with isoflurane (Shanghai Yuyan Scientific Instruments Co., Ltd.), mice were placed in a supine position on a special black pad for laser Doppler imaging (MoorLDI2, Moor Instruments Inc, US). The feet were fixed with a double-sided adhesive to ensure that they were upward and in a left-right symmetrical position. The position of the scanning probe was adjusted abovementioned the mice, at a distance of about 40 cm, to ensure that the scanning area included the whole hindlimbs. Laser Doppler imaging was then used to determine the blood flow in the hindlimbs of mice on the day of the operation and days 7 and 14 after the operation.

### 2.4. Sample Collection

Samples were collected 7-and 14-days postoperation. Blood samples were collected and centrifuged at 20267 g for 15 min at 4°C. The supernatant was then collected and immediately stored at −80°C for further use. Next, the rectus femoris of the left hindlimb was isolated: half of it was immediately stored at −80°C for further analysis, and the other half was stored in 4% paraformaldehyde for 24 h.

### 2.5. Enzyme-Linked Immunosorbent Assay (ELISA)

The levels of interleukin (IL)-17A and IL-21 in the serum of mice were determined by ELISA. Notably, IL-17A (cat. no. F07936, Shanghai Rigorous Biotechnology Co., Ltd) and IL-21 (cat. no. F01224, Shanghai Rigorous Biotechnology Co., Ltd) kits were used according to the manufacturer's instructions. Briefly, different concentrations of standard 50 *μ*L were added into the standard pore of the enzyme label plate, i. e., 10 *μ*L of mouse serum and 40 *μ*L of sample diluent. Next, 100 *μ*L horseradish peroxidase (HRP)-labeled antibody was added to each well, followed by blocking the reaction hole with a sealing membrane and incubation at 37°C for 60 min. The liquid was then removed, and the plate was dried using absorbent paper and washed five times with the washing machine. Next, 50 *μ*L of substrate A and 50 *μ*L of substrate B were added to each well, and these were incubated in the dark at 37°C for 15 min. Finally, a 50 *μ*L termination solution was added, and the optical density (OD) values of each well were measured at 450 nm within 15 min. The standard curve was generated according to the OD of the standard protein. Unknown sample protein concentrations could then be obtained from this standard curve.

### 2.6. Western Blot

Total 30 mg of muscle tissues were weighed, immersed into RIPA lysis buffer (Beyotime Institute of Biotechnology), and grinded in a tissue crushing instrument. After lysing for 30 minutes on ice, the muscle tissue was centrifuged at 120 000 rpm/min for 20 min, and then the supernatant was collected. The protein concentration in the supernatant was detected using the BCA protein detection kit (cat. no. P0010; Beyotime) according to the manufacturer's protocol. Total 30 *μ*g of protein samples in each group were added to a 10% sodium dodecyl sulfate-polyacrylamide gel for electrophoresis and transferred to methanol-activated polyvinylidene difluoride (PVDF) membranes (IPVH00010; Millipore). After blocking with 5% skim milk or bovine serum albumin (BSA) at room temperature for 1 h, the membranes were incubated with *β*-tubulin (1 : 4000; cat. no. A01030; Abbkine), IL-17A (1 : 4000; cat. no. ab79056; Abcam), JAK2 (1 : 1000; cat. no. D2E12; CST), p-JAK2 (1 : 1000; cat. no. Y1007/1008; CST), STAT3 (1 : 1000; cat. no. D1B2J; CST), and p-STAT3 (1 : 1000; cat. no. Y750; CST) at 4°C overnight. On the next day, membranes were washed with 1 × TBST buffer for three times for 10 min each and incubated with HRP-labeled goat anti-rabbit secondary antibody (1 : 4000; cat. no. A21020 Abbkine) or goat anti-mouse secondary antibody (1 : 4000; cat. no. A21010; Abbkine) at room temperature for 1 h. After incubation of secondary antibodies, membranes were washed with 1 × TBST buffer for three times for 5 min each. Finally, protein bands were visualized using an enhanced chemiluminescence reagent (cat. no. HP5002; Noble Biology Co., Ltd). Image J software was used to quantify the gray values of each band and the relative protein expression was calculated based on the gray values. The expression of IL-17A protein in each group was analyzed with *β*-tubulin as control. p-JAK2 and p-STAT3 were compared between the groups using JAK2 and STAT3 as controls.

### 2.7. Hematoxylin and Eosin (H&E) Staining

Muscle tissues were fixed in 4% paraformaldehyde solution for more than two days, and then the paraformaldehyde was discarded, and tissues were rinsed for 1 h. After conventional dehydration, tissues were embedded in paraffin, followed by sectioning (4 *μ*m-thick sections) using the microtome (Leica, RM2016) and staining with hematoxylin and eosin (H&E). Next, the sections were roasted, dewaxed with xylene I and xylene II for 10 min each, and then dewaxed with gradient alcohol for 5 min. Finally, an optical microscope was used to observe muscle texture changes after conventional dehydration and transparent neutral gum sealing.

### 2.8. Masson Staining

Paraffin sections were dewaxed to water, followed by staining the nuclei with hematoxylin. Next, 1% hydrochloric acid was used for differentiation treatment, and the sections were then rinsed back to blue. The sections were subjected to Lichun red staining and then quickly rinsed with distilled water. Additionally, phosphomolybdic acid treatment was performed, followed by aniline blue staining and 1% acetic acid differentiation treatment. Finally, muscle texture changes were observed under an optical microscope after conventional dehydration and transparent neutral gum sealing.

### 2.9. Statistical Analyses

All statistical analyses were performed using GraphPad Prism 9.0 (GraphPad Software, Inc.), and all data were expressed as (± standard error of the means [SEM]). One-way analysis of variance (ANOVA) was used to compare differences among multiple groups. All experiments were performed at least three times. A *P* of <0.05 was considered statistically significant.

## 3. Results

### 3.1. Effect of RJQM Treatment on the Blood Flow of Ischemic Lower Limbs

**Figure 1:**
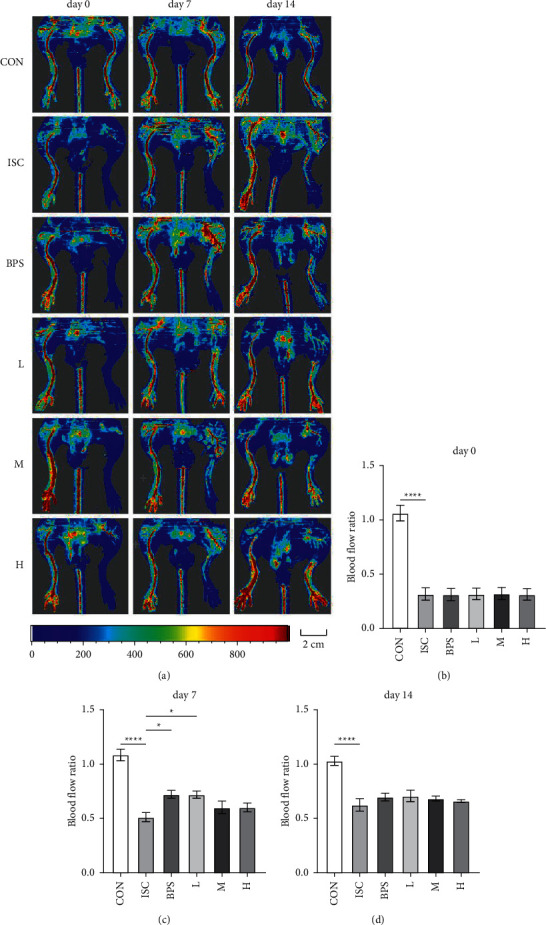
Changes in hindlimb blood flow in mice. (a) Representative blood flow images of hindlimbs of mice on days 0, 7, and 14. (b) A comparison of blood flow values of the left and right hindlimbs between the groups on the same day of the operation. (c) A comparison of blood flow values of the left and right hindlimbs between the groups on the 7^th^ day. (d) A comparison of blood flow values of the left and right hindlimbs between the groups on the 14^th^ day. Scale bar = 2 cm; CON, control group; ISC, ischemic group; BPS, sodium beraprost; L low dose of RJQM; M medium dose of RJQM; and H high dose of RJQM. ^*∗*^*P* < 0.05, ^*∗∗∗∗*^*P* < 0.0001. The data are expressed as ± standard error of the mean (SEM) (*n* = 10 in each group).

Laser Doppler imaging was used to monitor blood flow in the hindlimbs of mice (Figure 1(a)). On the day of the operation, the arterial blood vessels of both lower limbs were clearly visible, filled with blood, and had abundant blood flow. The femoral artery of the left hindlimb was then ligated and disconnected in the ISC and treatment groups. After the operation, the blood flow of the affected limb dropped sharply to <30% of the normal side (*P* < 0.0001), indicating the successful establishment of the ISC model (Figure 1(b)). Seven days after the operation, the blood flow in the hindlimbs of mice in the control group was intact, with a ratio of about 1. In the ISC group, the blood flow ratio of the healthy side recovered to 0.5 (*P* < 0.01), which was comparable to the control group. Compared to the ISC group, the blood flow ratio of the BPS and L groups recovered to about 0.7 (*P* < 0.05, *P* < 0.05), whereas that of the M and H groups recovered to about 0.6 (Figure 1(c)). Moreover, the blood flow ratio of the ISC group returned to 0.6 after 14 days of treatment, whereas the blood flow ratio of each treatment group returned to about 0.7 (Figure 1(d)), which was slightly higher than that of the ISC group, but the difference was not statistically significant.

### 3.2. Effect of RJQM Treatment on the Inflammatory Response of Rectus Femoris

**Figure 2:**
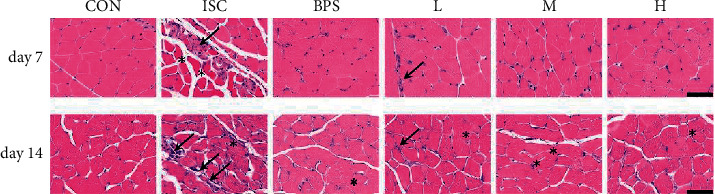
The inflammatory manifestations were evaluated with H&E staining. The representative images of the rectus femoris of the left hindlimb of the experimental group and the control group were treated for 7 and 14 days. Inflammatory cell infiltration (↙); Muscle fiber swelling (^*∗*^). Magnifications: ×400, Scale bar = 60 *μ*m. CON, control group; ISC, ischemic group; BPS, sodium beraprost; L low dose of RJQM; M medium dose of RJQM; and H high dose of RJQM.

H&E staining was used to observe morphological changes and inflammatory infiltration in muscle tissues. Results showed that on the 7^th^ day of drug treatment, most muscle fibers in the ISC group were irregular in shape, disordered in arrangement, swollen, and uneven in size, with widened intercellular space and inflammatory cell infiltration between muscle fibers compared to the CON group (Figure 2). After 14 days of treatment, the infiltration of inflammatory cells was increased. Specifically, the infiltration of inflammatory cells between muscle fibers was alleviated to varying degrees in the BPS group and all RJQM treatment groups compared to the ISC group.

### 3.3. Effect of RJQM Treatment on the Level of Fibrosis of Rectus Femoris

**Figure 3:**
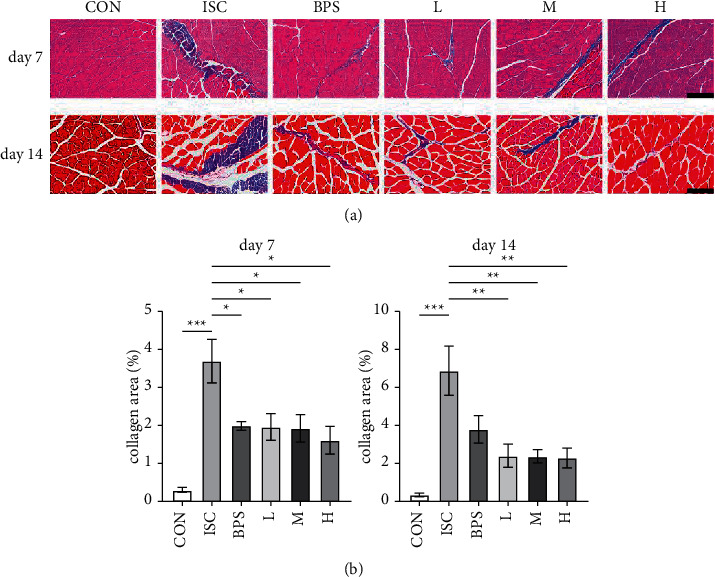
Evaluation of fibrosis according to Masson staining. (a) Representative images of rectus femoris of the left hindlimb in the experimental group and the control group on the 7^th^ and 14^th^ days. (b) Quantification of the percentage area of collagen-positive cells (blue) in each image. Magnifications: ×200, Scale bar = 100 *μ*m. CON, control group; ISC, ischemic group; BPS, sodium beraprost; L low dose of RJQM; M medium dose of RJQM; and H high dose of RJQM. ^*∗*^*P* < 0.05, ^*∗∗*^*P* < 0.01, ^*∗∗∗*^*P* < 0.001. The data expressed as ± standard error of the mean (SEM) (*n* = 3 in each group).

Muscle sections were subjected to Masson trichrome staining (Figure 3(a)), and the percentage area of collagen-positive tissue (blue in color) was quantified (Figure 3(b)). On the 7^th^ day, the content of collagen in the ISC group significantly increased compared to the control group (*P* < 0.001), whereas the content of collagen in the BNP group and all RJQM treatment groups decreased compared to the ISC group (*P* < 0.05, *P* < 0.05, *P* < 0.05, *P* < 0.05). After 14 days, the collagen fibers in the ISC group continued to increase (*P* < 0.001), and the percentage of collagen staining in the L, M, and H groups was decreased compared to the ISC group (*P* < 0.01).

### 3.4. Effect of RJQM Formula on IL-17A and IL-21 Levels in Serum

**Figure 4:**
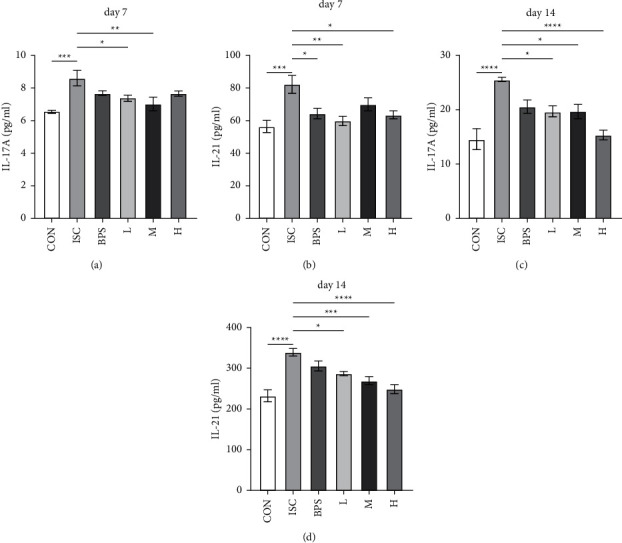
Serum levels of IL-17A and IL-21 in mice. (a) and (b) The levels of IL-17A and IL-21 in the serum of mice in each group were observed after seven days. (c) and (d) the levels of IL-17A and IL-21 in the serum of mice after 14 days. CON, control group; ISC, ischemic group; BPS, sodium beraprost; L low dose of RJQM; M medium dose of RJQM; and H high dose of RJQM. ^*∗*^*P* < 0.05, ^*∗∗*^*P* < 0.01, ^*∗∗∗*^*P* < 0.001, ^*∗∗∗∗*^*P* < 0.0001. The data are expressed as ± standard error of the mean (SEM) (*n* = 6 for each group).

We explored the expression of IL-17A and IL-21 in the serum of mice. Results obtained on the 7^th^ day showed that the expression of IL-17A (Figure 4(a)) in the ISC group was significantly upregulated compared to the control group (*P* < 0.001). However, the expression of IL-17A was reduced in the L (*P* < 0.05) and M groups (*P* < 0.01) compared to the ISC group. On the other hand, the expression of IL-21 (Figure 4(b)) in the ISC group was also significantly upregulated compared to the CON group (*P* < 0.01). The BPS (*P* < 0.05), L (*P* < 0.01), and H groups (*P* < 0.05) had significantly reduced expression of IL-21 compared to the ISC group (*P* < 0.01).

See Figure 4(c) shows the results after 14 days. The expression of IL-17A in the ISC group was significantly upregulated compared to the CON group (*P* < 0.0001), whereas the expression of IL-17A in the L (*P* < 0.05), M (*P* < 0.05), and H groups (*P* < 0.0001) was reduced compared to the ISC group (Figure 4(d)). Additionally, the expression of IL-21 in the ISC group was significantly upregulated compared to the CON group (*P* < 0.0001). It was found that the L (*P* < 0.05), M (*P* < 0.001), and H groups (*P* < 0.0001) could reduce the expression of IL-21 in a dose-dependent manner.

Noteworthy, the statistics rule of other inflammatory factors, including IL-2, IL-3, IL-4, IL-6, IL-10, IL-22, interferon (IFN)-*γ*, and transforming growth factor (TGF)-*β*, could not be obtained in each group by detecting their expression levels (Figure S1).

### 3.5. Effect of RJQM Treatment on the JAK2/STAT3, IL-17A Signaling Pathway

**Figure 5:**
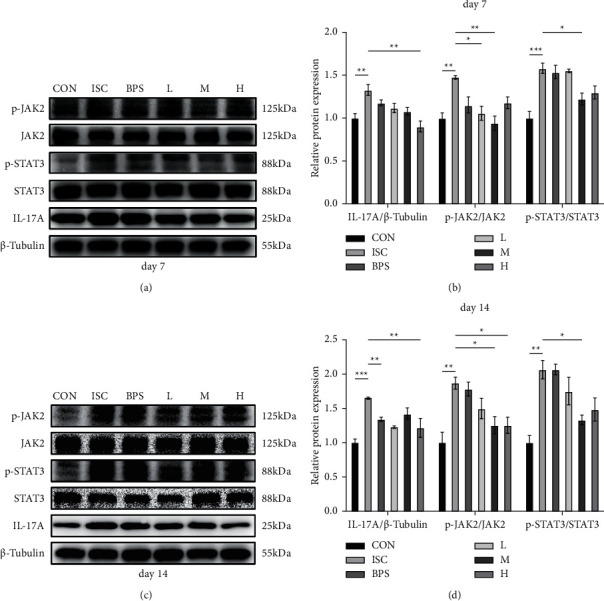
Expression of JAK2, STAT3, and IL-17A in the rectus femoris. (a and c) Representative western blots of JAK2, STAT3, and IL-17A protein levels in the rectus femoris after 7 and 14 days, with *β*-tubulin as control. (b and d) An average gray value of JAK2, STAT3, and IL-17A protein levels in the rectus femoris on the 7^th^ and 14th day. CON, control group; ISC, ischemic group; BPS, sodium beraprost; L low dose of RJQM; M medium dose of RJQM; and H high dose of RJQM. ^*∗*^*P* < 0.05, ^*∗∗*^*P* < 0.01, ^*∗∗∗*^*P* < 0.001. The data are expressed as ± standard error of the means (SEM) (*n* = 3 for each group).

Furthermore, we explored the expression of JAK2, STAT3, and IL-17A proteins in muscle tissues.  Figure 5(a) shows the results obtained on the 7^th^ day. It was found that the relative expression levels of p-JAK2 (*P* < 0.01), p-STAT3 (*P* < 0.001), and IL-17A (*P* < 0.01) proteins were significantly upregulated in the ISC group compared to the CON group (Figure 5(b)). After the treatment, the expression levels of p-JAK2, p-STAT3, and IL-17A in the BPS, L, M, and H groups were downregulated to varying degrees.

Specifically, the expression of p-JAK2 protein was significantly reduced in the L (*P* < 0.05) and M (*P* < 0.01) groups compared to the ISC group. The expression of p-STAT3 protein was only significantly decreased in the M group (*P* < 0.05), whereas the expression of IL-17A protein was reduced in the H group (*P* < 0.01).  Figure 5(c) shows the results obtained after 14 days. The expression of IL-17A (*P* < 0.001), p-JAK2 (*P* < 0.01), and p-STAT3 (*P* < 0.01) proteins was significantly upregulated in the ISC group compared to the CON group (Figure 5(d)). Compared to the ISC group, the expression of p-JAK2 protein was inhibited in the M (*P* < 0.05) and H (*P* < 0.05) groups, whereas the expression of p-STAT3 protein was inhibited in the M group (*P* < 0.05). Moreover, the L (*P* < 0.01) and H (*P* < 0.01) groups could significantly inhibit the expression of IL-17A protein compared to the ISC group.

## 4. Discussion

Herein, results showed that RJQM could promote the recovery of blood flow to a certain extent and relieve inflammation and fibrosis in the hindlimb ischemic rectus femoris. At the same time, it was found that RJQM could reduce the level of inflammatory factors in a process that may be associated with the inhibition of the JAK2/STAT3 signaling pathway.

Blood flow recovery in the ischemic hindlimb of mice depends on the establishment of collateral circulation. Considering that the anti-angiogenic environment generated by inflammation or oxidative stress is associated with the disease [15], inflammatory cells can damage angiogenesis and perfusion recovery of C57BL/6 ischemic muscles [16]. Results obtained in this study demonstrated an increase in hindlimb blood flow on the 7^th^ day after RJQM treatment, especially in the L group. At 14 days, the blood flow was higher in all treatment groups compared to the ischemia group, but there was no significant difference between the groups. This could be attributed to the fact that previous studies have shown that C57BL/6 mice have a strong tolerance to ischemia and can quickly recover from ischemia [17, 18]. Therefore, by the 14^th^ day, the blood flow had reached a certain level on its own. Although RJQM treatment could promote blood flow recovery, it could not compensate for the damage caused by surgery; thus, there was no significant difference with the ischemia group. Notably, the efficacy of the L group was better than that of the M and H groups. These results suggest that RJQM can promote the recovery of blood flow in ischemic hindlimbs of mice to some extent, but the specific mechanism should be elucidated using further studies.

A previous study has found that acute limb ischemia can cause muscle damage, but the damage depends on the severity of ischemia [19]. Ischemia and hypoxia of muscle tissue can lead to an inflammatory response in muscle fiber injury [20], which can, in turn, lead to tissue fibrosis. Several studies have revealed that the ischemic rectus femoris exhibits obvious inflammatory cell infiltration and muscle fiber necrosis [21, 22], increased collagen content and increased fibrosis level in the hindlimb mice ischemia model [5]. In this study, the ligation and disconnection of the femoral artery on the left hindlimb caused inflammation of the rectus femoris, which was consistent with the muscle inflammatory response in the model experiment. After the treatment for 7 and 14 days, the damage in each treatment group was alleviated, and the infiltration of inflammatory cells was improved. Additionally, RJQM treatment decreased the collagen content in the rectus femoris of the ischemia model, and the level of fibrosis was lower. Treating for 14 days had a more obvious effect on muscle fibrosis compared to treating for seven days, indicating that RJQM could better alleviate the inflammatory reaction and fibrosis in ischemic muscle tissue with time. This is consistent with findings of previous studies on bone marrow mesenchymal stem cell transplantation [22] and the increase in the release of alkaline fibroblast growth factor [5], which can reduce the inflammatory cell infiltration and fibrosis in the hindlimb muscle of mice with ischemia. However, the difference between inflammatory and blood flow changes is inconsistent, which is in line with a previous study that has found that ischemia-induced rectus femoris injury did not completely correspond to muscle blood flow [19].

Atherosclerosis is considered a chronic nonspecific inflammatory disease mediated by multiple immune cells. Notably, immune cells are abundant in atherosclerotic lesions and produce cytokines, especially proinflammatory cytokines [23]. For example, Th cells can secrete inflammatory mediators involved in local vascular intimal inflammation. IL-17 A is a proinflammatory cytokine mainly secreted by Th17 cells [24]. Additionally, clinical studies have found that peripheral blood mononuclear cells (PBMCs) in atherosclerosis patients release increased IL-17, especially in severe patients [25]. In atherosclerosis, endothelial dysfunction and vascular inflammation are reversed by lowering IL-21 [26]. This study showed that RJQM could reduce the levels of IL-17 A and IL-21 in the serum of mice, but it had no effect on some inflammatory factors, such as IL-2, IL-3, IL-4, IL-6, IL-10, IL-22, INF-*γ*, and TGF-*β*. However, further studies should be conducted to elucidate the specific mechanism of RJQM action.

The JAK2/STAT3 signaling pathway is one of the three classical inflammatory signaling pathways that can regulate the main mechanism of the inflammatory response [27]. One study has reported that it is closely associated with atherosclerosis occurrence and development [8]. Notably, the JAK/STAT pathway can be activated by proinflammatory cytokines, thereby leading to phosphorylation of STAT transcription factors and inflammatory response. Moreover, IL-17A and IL-21 can activate JAK and STAT family signal transduction, consequently playing a key role in inflammatory diseases [28]. This study found that RJQM can inhibit the expression of JAK2/STAT3 proteins, which suggests that RJQM might alleviate inflammation caused by ischemia and hypoxia through the JAK2/STAT3 signaling pathway. However, given that there was a lack of inhibitor interference, further research should be conducted to confirm this finding.

BPS, a first-line drug for clinical treatment of ASO, has strong antiplatelet aggregation and vasodilation properties. Clinical trials have proven that a certain dose of BPS has an effect on improving ASO symptoms and prognosis [2]. The drug can significantly reduce the inflammatory response in arteries and improve the blood supply to the affected limbs and has an obvious curative effect on ASO patients [7]. Therefore, BPS was selected as the positive drug in this study. Herein, results showed that BPS could promote the recovery of blood flow in ischemic lower limbs and slow down inflammation and fibrosis of the rectus femoris. However, RJQM could significantly reduce the level of inflammatory factors and expression of related proteins compared to BPS, suggesting that the anti-inflammatory effect of RJQM is superior to BPS.

## 5. Conclusions

In conclusion, this study showed that RJQM could alleviate ASO by reducing the inflammatory response by inhibiting the JAK2/STAT3 signaling pathway. These findings provide a scientific basis for further clinical application of RJQM. However, there are some limitations to this study. First, the detailed effect mechanism needs to be further investigated in cell experiments. Second, clinical trials requiring long-term and large-scale collection of ASO patients will be carried out to obtain more experimental data. All of the abovementioned studies will contribute to the establishment of the theoretical foundation for the treatment of ASO with RJQM.

## Data Availability

The data used to support the findings of this study are available from the corresponding author upon request.

## Supplementary Materials

Supplementary Materials See Figure S1 in the Supplementary Material for more inflammatory factors analysis.
