# Supplementary material for: Ruan Jian Qing Mai Recipe Inhibits the Inflammatory Response in Acute Lower Limb Ischemic Mice through the JAK2/STAT3 Pathway
Source: Evid Based Complement Alternat Med. 2022 Aug 18;2022:2481022. doi: 10.1155/2022/2481022 (PMC9410777; doi:10.1155/2022/2481022)
Supplement: Supplementary Materials — See Figure S1 in the Supplementary Material for more inflammatory factors analysis. [file 2481022.f1.pdf]

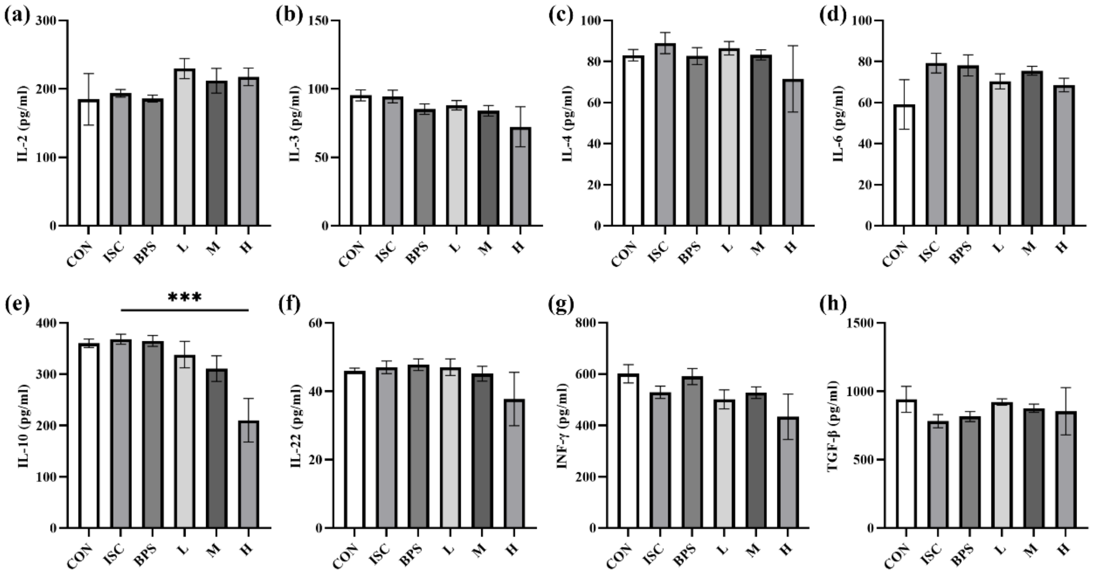

Figure S1. Serum levels of other inflammatory factors in mice. (a)-(h) The levels of IL-2, IL-3, IL-4, IL-6, IL-10, IL-22, INF-γ, and TGF-β in serum of mice in each group were observed after 14 days. CON, control group; ISC, is chemic group; BPS, sodium beraprost; L, low dose of RJQM; M, medium dose of RJQM; and H, high dose of RJQM. * * * $P < 0.001$. The data expressed as $\overline{X} \pm$ SEM (n = 6 for each group).
